# Targeted memory reactivation elicits temporally compressed reactivation linked to spindles

**DOI:** 10.1162/IMAG.a.1123

**Published:** 2026-02-03

**Authors:** Mahmoud E.A. Abdellahi, Martyna Rakowska, Matthias S. Treder, Penelope A. Lewis

**Affiliations:** School of Psychology, Cardiff University Brain Research Imaging Centre (CUBRIC), Cardiff, United Kingdom; Faculty of computers and artificial intelligence, Cairo University, Giza, Egypt; School of Engineering, Computer Science department, University College London (UCL), London, United Kingdom; School of Computer Science and Informatics, Cardiff University, Cardiff, United Kingdom.

**Keywords:** targeted memory reactivation, NREM sleep, memory reactivation, classification, spindles, compressed reactivation

## Abstract

Memories reactivate during sleep, however the properties of such reactivation and its relationship to subsequent memory performance are not well understood. Here, we set out to examine memory reactivations associated with a serial reaction time task (SRTT). 48 human participants performed the SRTT and then slept in the lab while we deliberately induced reactivation in Slow Wave Sleep (SWS) using a Targeted Memory Reactivation (TMR) design. We detected reactivation after TMR cues using multiclass classification that adapted to sleep data by using sleep activity for training and wake activity for testing. We then examined the temporal properties of reactivation in relation to behavioral performance and sleep spindles. The observed reactivation was 3 to 20 times faster than waking activity. Finally, reactivation was more frequently observed in trials with high sigma power, supporting the idea that sleep spindles are associated with memory reactivation during sleep. These findings bring us closer to understanding the characteristics of human memory reactivation after TMR and provide evidence for the positive relationship between the detectability of reactivation and memory consolidation.

## 1 Introduction

We spend around one third of our lives asleep. During sleep, the brain is busy processing memories through replay or reactivation which is essential for memory consolidation (Diekelmann & Born, 2010; Rasch & Born, 2013; Squire et al., 2015).

The active system consolidation (ASC) hypothesis (Diekelmann & Born, 2010) suggests that sleep is not merely a passive shelter for memories against interference. Instead, newly encoded memories repeatedly reactivate during slow wave sleep (SWS) and this strengthens those memories in an ongoing process of memory consolidation. The ASC model (Rasch & Born, 2013) proposes a dialogue between neocortex and hippocampus in which slow oscillations (SOs) drive reactivation of hippocampal memories, with accompanying sharp wave ripples that are carrying reactivations nested into thalamo-cortical spindles. The model also suggests that spindles prime the cortex for reactivation-related plasticity by stimulating calcium influx into the dendrites of cortical pyramidal cells.

A technique called targeted memory reactivation (TMR) can be used to manipulate reactivation in sleep. In TMR, cues such as odors, sounds, or electrical shocks are associated with the learned material as a result of being presented during memory encoding or retrieval. Cues are then re-delivered during subsequent sleep and thereby thought to reactivate the cued memory (Hennevin & Hars, 1987). In humans, several studies have shown the benefits of TMR during sleep on memory consolidation for both declarative (Cairney et al., 2014; Fuentemilla et al., 2013; Rasch et al., 2007; Rudoy et al., 2009) and non-declarative memories (Antony et al., 2012; Schönauer et al., 2014). Memory reactivation elicited via TMR can be detected using multivariate pattern classifiers and similarity analyses (Abdellahi et al., 2023b; Belal et al., 2018; Cairney et al., 2018; Schreiner et al., 2018; Wang et al., 2019). However, despite extensive research in the area, there are still a lot of gaps in our understanding of the characteristics of cued reactivation. Are such reactivations exact clones of wake activations, or do they differ in shape or duration? How do sleep spindles relate to memory reactivation? And how does reactivation detection relate to consolidation? Here, we set out to answer these questions and thereby gain a better understanding of memory reactivation and TMR.

In rats, memory replay during NREM sleep has been shown to have different temporal characteristics compared to wake, as it occurs from 10 to 20 times faster (Ji & Wilson, 2007; Lee & Wilson, 2002; Nádasdy et al., 1999). In wake, offline replay is thought to occur from 6 to 7 times faster than the actual task (Euston et al., 2007). The firing activity of individual neurons measured in non-human studies gives clear evidence of compression, while EEG in humans offers high temporal resolution but lacks the spatial resolution necessary for direct analysis of temporal compression. To address this challenge, we developed an approach that systematically rescales variable-duration sleep windows to match wake trial length, testing whether optimal pattern similarity occurs at specific compression ratios. This method allowed us to identify temporal compression by detecting when temporally rescaled sleep neural patterns exhibited enhanced feature correspondence with their wake counterparts, thereby providing evidence for compressed memory reactivation at the EEG level.

The reactivation-spindle connection is supported by Cairney and colleagues who showed that spindles mediate reactivation in human NREM sleep (Cairney et al., 2018). Additionally, a significant post-cue reactivation was observed in trials with high post-cue power in the spindle band (Wang et al., 2019), while enhancing spindles led to more consolidation (Lustenberger et al., 2016; Ngo et al., 2013). It has also been shown that hippocampal sharp-wave ripples are nested in the troughs of spindles (Staresina et al., 2015). Our current study investigated how sleep spindles relate to reactivation.

**Fig. 1.**
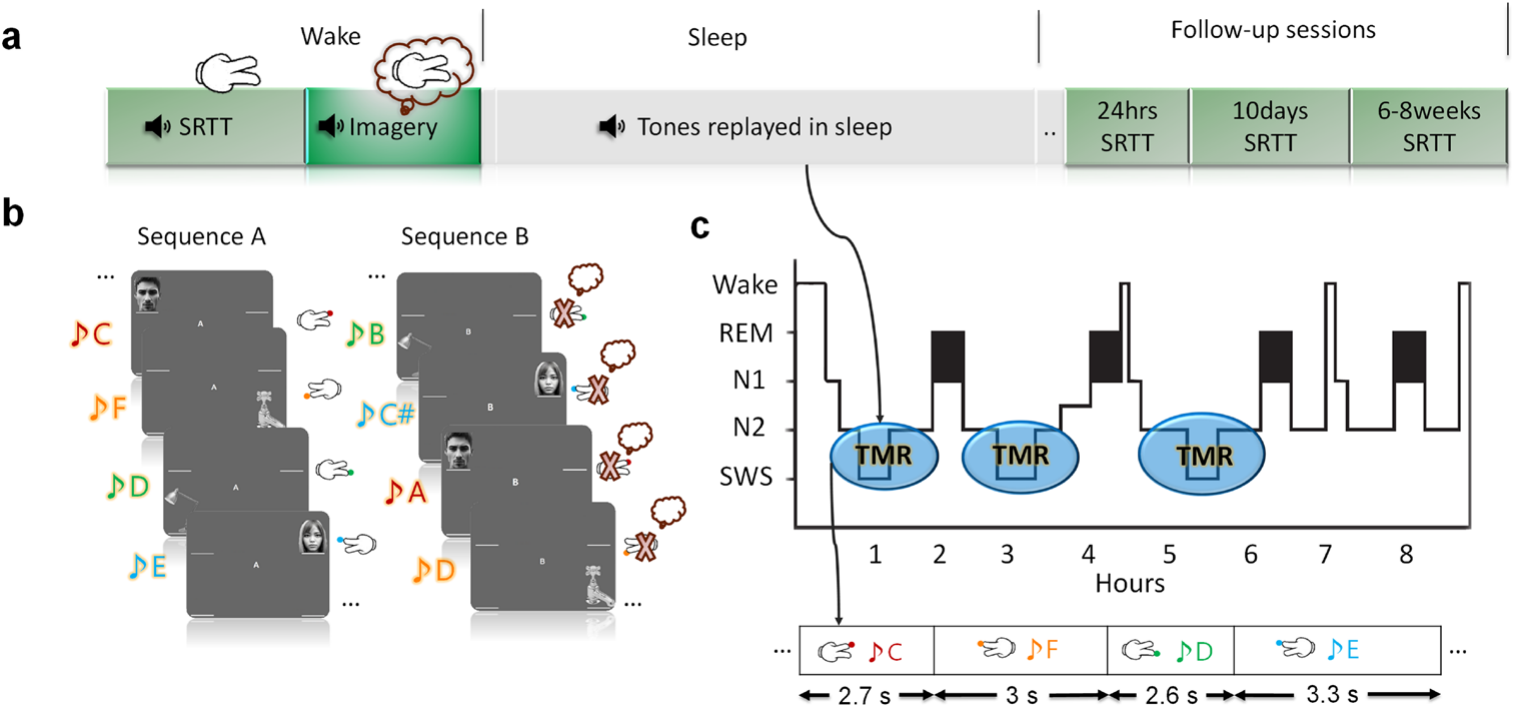
Study design. (a) We analyzed sleep and wake data from 48 participants. Participants first performed a serial reaction time task (SRTT), followed by a motor imagery task, both with the EEG headcaps on. Subsequently, they went to sleep and TMR was carried out in NREM sleep, as shown in panel c. After that, the participants were tested on the SRTT in three follow-up sessions. (b) In the SRTT, four images are presented in two different sequences. Each image is accompanied by a specific tone (different for each sequence) and requires a specific button to be pressed. In the imagery task, participants view the same sequences of images but only imagine they are pressing the buttons without any actual movements. This motor imagery task served as a clean template for characterizing wake pattern and was later used in classification. (c) TMR took place in NREM sleep with jittered intertrial intervals between 2500 ms and 3500 ms. Each sequence was followed by a 20-second pause.

We used a serial reaction time task (SRTT), which is known to be sleep sensitive (Born & Wilhelm, 2012; Spencer et al., 2006) and also sensitive to TMR in non-REM sleep (Cousins et al., 2014, 2016) (see Fig. 1 for experimental design and Supplementary Fig. S1) to investigate the characteristics of cued reactivation. In the SRTT, participants saw an image on one of four quadrants of the screen and simultaneously heard a distinct sound that was associated with that image during encoding. We then distinguished between reactivation of four distinct memories after TMR cues by directly relating wake and sleep EEG in 48 participants. We introduce a classification pipeline in SWS that uses sleep activity for the training of classifiers and wake activity for testing, which allows classifiers to adapt to sleep features that are related to reactivation when adjusting their weights.

## 2 Methods

### 2.1 Participants

We collected EEG and behavioral data from human participants (n = 48) (25 females, age mean ±SD: 19.9 ±1.4; 23 males, age: 20.8 ±2.1). The number of participants was reduced from 56 as some of them were excluded for technical problems during recording of sleep. Participants completed the SRTT before sleep and during three follow-up sessions, the first one was after the night of stimulation (24 hours), the second after 10 days later, and eventually the final session after 6 to 8 weeks. All participants were right-handed with no prior knowledge of the SRTT. All participants had normal or corrected-to-normal vision, normal hearing, and no history of physical, psychological, neurological, or sleep disorders. Responses in a pre-screening questionnaire reported no stressful events and no travel before commencing the study. Participants did not consume alcohol or caffeine in the 24 hours prior to the study or perform any extreme physical exercise or nap. This study was approved by the School of Psychology, Cardiff University Research Ethics Committee, and all participants gave written informed consents.

### 2.2 Experimental design

Participants completed the SRTT adapted from (Cousins et al., 2014). Participants learned two 12-item sequences, A and B (A: 1 2 1 4 2 3 4 1 3 2 4 3 and B: 2 4 3 2 3 1 4 2 3 1 4 1). Sequences had been matched for learning difficulty; both contained each item three times. Sequences were presented in blocks and each block contained three repetitions of a sequence. The blocks were interleaved so that a block of the same sequence was presented no more than twice in a row. There were 24 blocks of each sequence (48 blocks in total), and each block was followed by a pause of 15 seconds during which feedback on reaction time (RT) and error-rate were presented. After the 48 blocks of sequences A and B, participants performed four blocks of random sequences. They contained the same visual stimuli, two of these blocks were paired with the tone group of one sequence (reactivated in sleep), and the other two with the tone group of the other sequence (not reactivated). Participants were aware that there were two twelve-item sequences, and each sequence was indicated with ‘A’ or ‘B’ appearing centrally on the screen, but participants were not asked to learn the sequences explicitly. Counterbalancing across participants determined whether sequence A or B was the first block, and which of the sequences was reactivated during sleep. Each sequence was paired with a group of pure musical tones, either low tones within the 4th octave (C/D/E/F) or high tones within the 5th octave (A/B/C#/D). These tone groups were counterbalanced across sequences. For each trial, a 200 ms tone was played, and at the same time a visual cue appeared in one of the corners of the screen. The location indicated which key on the keyboard needed to be pressed as quickly and accurately as possible: 1 – top left corner = left shift; 2 – bottom left corner = left Ctrl; 3 – top right corner = up arrow; and 4 – bottom right corner = down arrow. Participants were instructed to keep individual fingers of their left and right hand on the left and right response keys, respectively. Visual cues were neutral objects or faces, used in previous studies (Cousins et al., 2014), which appeared in the same position for each sequence (1 = male face, 2 = lamp, 3 = female face, 4 = water tap). After responding to the visual cues with the correct key press, an 880 ms inter-trial interval followed.

After completion of the SRTT, participants were asked to do the same task again, but were instructed to only imagine pressing the buttons. Motor imagery (IMG) consisted of 30 interleaved blocks (15 of each sequence), presented in the same order as during the SRTT. Each trial consisted of a 200 ms tone and a visual stimulus which was presented for an 880 ms followed by a 270 ms inter-trial interval. There were no random blocks during the imagery task, and no performance feedback was presented during the pause between blocks. We collected the SRTT data during three sessions after the stimulation night, with one the next day (24 hours) after performing the task and spending the night in the lab, the second one after 10 days, and the third after 6 to 8 weeks. During the night of stimulation, cues were presented during NREM sleep with the continuous supervision of experiments and data scored as N3 was the one included in the analysis. Inter-trial intervals were jittered between 2500 ms and 3500 ms, as demonstrated in Figure 1. Stimulation was paused with any signs of arousals until the experimenters observe approximately three 30-second epochs with stable NREM sleep. In the follow-up sessions (24 hours, 10 days, and 6 to 8 weeks) after the task, participants were asked to perform the SRTT again. Eventually, in the last session, they were asked if they remember the locations of images of the two sequences in order to see if one sequence is recalled better than the other one. Motor imagery data set of each participant was used to classify the brain activity without movement artifacts.

### 2.3 Behavioral improvement

We measured the behavioral improvement after sleep in three different sessions: the first was after sleep, the second after 10 days, and the third after 6–8 weeks. Some participants were excluded from the analysis because they dropped out and did not come to the follow-ups; thus, the number of participants in this analysis was 41 participants. We were interested in the aggregated effect of TMR across these sessions. For every session, all 24 blocks containing the reaction times for a sequence were aggregated and the blocks with the best performance among them were kept based on the 95 percentiles of performance values. Thus, the fastest 5 percentiles of data were used from every session and the median of post-sleep sessions was calculated. The same procedure was conducted for pre-sleep session where the fastest 5 percentiles of blocks were used as the pre-sleep performance measure. Afterward, we determined the improvement as (pre-sleep–post-sleep); thus, a high value would reflect big improvement. We then tested for the difference between the improvement for the reactivated and the non-reactivated sequence using a Wilcoxon signed-rank test. This approach of focusing on the best blocks parallels the methodological approach proposed by Ribeiro (Pereira et al., 2015), who argued that selecting peak performance (‘best trials’) provides a more valid estimate of motor skill consolidation. Here, we extend the same principle to different sessions to ensure comparability of peak performance across conditions.

### 2.4 The relationship between reactivation strength and memory consolidation

We performed a correlation analysis between the classification performance of reactivation and memory improvement after sleep. Memory improvement for each participant was measured as the difference between the reaction time of the un-cued and the cued sequence, which reflects the cueing benefit. To measure the relationship between reactivation and the direct cueing benefit, we used the follow-up session that came after sleep. The strength of memory reactivation was determined by the maximum classification CCR value for each participant. In this partial correlation, we controlled for the effects of the encoding session reaction times. Blocks of behavioral reaction times were aggregated into one value for each participant in the same way we calculated the behavioral improvement by keeping the fastest 5 percentiles of performance values and then taking their median.

### 2.5 EEG recording

The current study uses EEG from human participants. EEG was collected using 64 actiCap active electrodes with 62 channels on the scalp, including the reference electrode at CPz and ground electrode at AFz. Two electrodes were used on the left and right sides above and below the eyes for collecting electrooculography (EOG) signals and two electrodes on the right and left sides of chin for collecting the electromyography (EMG). Data were collected either at 500 Hz or 250 Hz and subsequently resampled to 200 Hz for all EEG analyses. Sound cues were delivered during NREM sleep. The data were re-referenced to the average of the mastoid channels (TP9, TP10), and the 58 EEG channels were then used in different analyses.

### 2.6 EEG cleaning

EEG cleaning consisted of filtering and outliers’ rejection based on statistical measures. EEG data were band-pass filtered (0.1 to 30 Hz) and centered. For sleep data, sleep was scored manually and only the trials in the epochs scored as N3 were used in this work. Afterward, we removed trials representing outliers based on statistical measures (variance, max, min) extracted for every trial and every channel. A trial is compared to all trials and considered as an outlier if it was higher than the third quartile + (the interquartile range *1.5) or less than the first quartile - (the interquartile range*1.5) in more than 25% of channels. If a trial was bad for <25% of channels, it was interpolated using neighboring channels with triangulation method in Fieldtrip. Furthermore, because the task is motor-related, we defined a number of channels around the motor area (C6, C4, C2, C1, C3, C5, CP5, CP3, CP1, CP2, CP4, and CP6) and a trial was rejected if it was bad on >25% of these channels otherwise bad channels were interpolated and the trial was kept.

### 2.7 Detecting memory reactivation with multivariate pattern classifiers

We used time-domain features in a multi-class classification pipeline with the EEG pattern from each of the four finger presses representing a class. Signals from the 58 EEG channels were smoothed using a moving averaging window of 100 ms, wherein each time point is replaced by the mean of the 100 ms around that point. This process was done for both sleep and wake data for each participant. Afterward, channels were reduced to principal components using sleep data (channels x time) from each participant through principal component analysis (PCA). PCA can be used to reduce dimensionality and reduce overfitting and has been adopted in several studies (Griffiths et al., 2021; Higgins et al., 2021; Peyrache et al., 2010; Schreiner et al., 2021; Tingley & Peyrache, 2020). Following this, we calculated the explained variance for each principal component (eigen value of a component / sum of all eigen values), we then sorted the principal components (PCs) based on the explained variances and kept the ones that contained 95% of the explained variance. Those PCs should be representing the dimensions in which the highest variance in the data exists and putative useful information. We then used the PCs and transformed both sleep and wake data which gave two transformed data sets containing PCs x time. Given the uncertainty of the timing of reactivation after our jittered cues and the possibility of temporal shifts in reactivation between participants, time points were concatenated and treated as observations to build one classification model. In this manner, we used all timepoints of sleep data to train one linear discriminant analysis (LDA) model (Blankertz et al., 2011). The trained LDA model was then applied to each time point after the cue in wake which yielded a classification accuracy at each wake time point. A classification output was then obtained from each participant, and the final output was compared to chance level of 0.25. The result was then corrected for multiple comparisons using cluster-based permutation in Fieldtrip (Oostenveld et al., 2011) and lively vectors (lv) (Abdellahi, 2022) which gave the same results. For cluster-based permutation, Monte Carlo was used with a sample-specific test statistic threshold = 0.05, permutation test threshold for clusters = 0.05, and 100,000 permutations. The correction window was the whole length of wake trial (1.15 second).

### 2.8 Compression and dilation of reactivation

A popular method for detecting the temporal compression of replay and used in the rodent literature is the template matching method. Generally, in template matching, a template is used from sleep episodes and this template is then slid on wake activity during maze navigation and a correlation coefficient is calculated which indicates the similarity of firing activity between the template and the window. This process was repeated for different scaling factors such that the windows were resized to smaller or longer sizes; the process was repeated to measure compression and dilation of replay. The spatial resolution of EEG signals is low; however, signals measured at different channels in sleep can be compared to the same channels in wake to infer their degree of similarity at different compression/dilation ratios. In our data, we adopted a classification-based method to detect compression/dilation of reactivation given the differences between EEG of multiple classes with TMR and continuous firing pattern and that our classifiers can adapt to sleep and detect their subtle features. We used different temporal ratios that represent the ratio between sleep trial duration and wake trial duration and for each ratio we evaluated the classification performance. For a given sleep trial duration, a temporal sliding window (shifted 10 ms each time) is used on sleep data and each window is resized to match the length of wake trial (illustration is provided in Supplementary Fig. S2). We adopted a similar approach of calculating the PCA and transformed the channels into PCs and we did not smooth the signals to keep the temporal information intact as smoothing could impact short effects. Both sleep and wake were transformed with the same PCs that were fitted on sleep data, so the features are projected to the same feature space. This implies that if there was an activity on specific PCs in sleep, the model will look at the same PCs in wake which will guarantee spatial alignment. Afterward, a classifier model was built using the concatenated features (PCs x timepoints) using sleep data and applied to wake data; this gave a classification performance for each compression/dilation ratio for each participant. Classification performance was then compared to chance level of 0.25 for each compression ratio using a Wilcoxon signed-rank test. We tested different temporal ratios that ranged from 20 faster to 2.2 slower reactivation compared to wake. Theoretically, we could check for faster compressions given that classification was significant for the 20 times faster reactivation. However, we did not go beyond 20 times because the sliding window in sleep will be shorter than 10samples (50 ms) and such very short window will not be reliable to resize and relate to wake to classify reactivation. In the meantime, we stopped at 2.2 slower reactivation because this matches the length of minimum sleep trial of 2.5 seconds divided by the length of wake trial of 1.15 seconds; thus, we stopped at this number to prevent any missing data points.

### 2.9 Spindle analysis and spindle-based reactivation predictors

We analyzed post-cue spindle activity to check if it relates to detected reactivation. We band-pass filtered our sleep data in the range [11 16]Hz using channel Cz and used the time duration [0 2.5]seconds; then, we used Hilbert transform. Afterward, we used the instantaneous magnitude and phase that resulted from the Hilbert transformation to get the power by taking the absolute value of the complex vector to get the magnitude and then squaring that magnitude to get the power. We then divided our trials into two groups for each participant: one with higher than median post-cue sigma power and the other with lower than median. A separate model was trained for each group and applied to all wake data from that participant.

EEG cleaning and other analyses (classification, compression/dilation, spindle analyses) were conducted with lively vectors (lv) (Abdellahi, 2022) toolbox developed by Mahmoud E. A. Abdellahi and it uses some functions from Fieldtrip (Oostenveld et al., 2011), MVPA-light (Treder, 2020), EEGLAB (Delorme & Makeig, 2004), and built-in Matlab functions.

## 3 Results

### 3.1 Elicited response after TMR cues

**Fig. 2.**
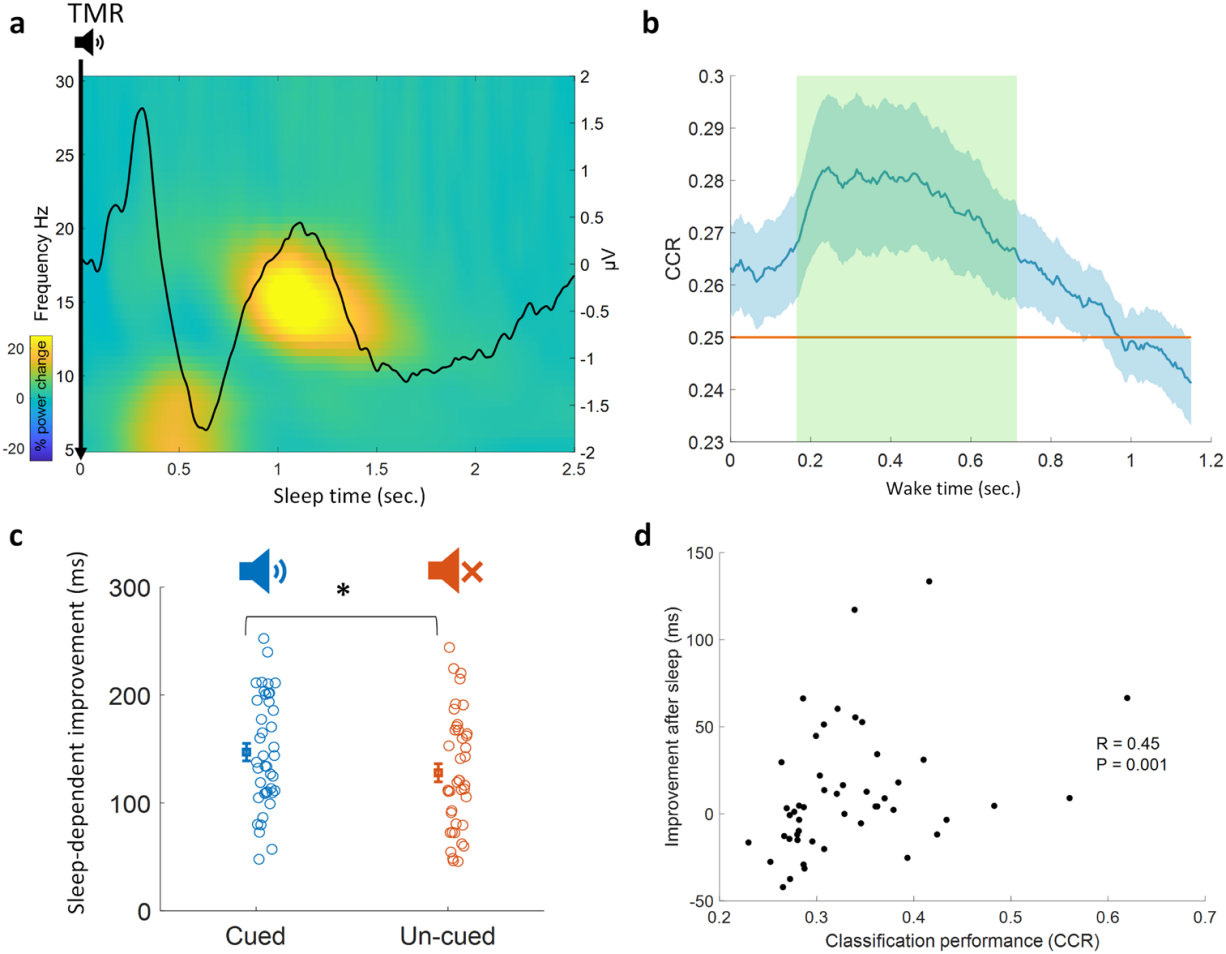
(a) Time-frequency and ERP analyses using sleep data from all participants (n = 48). Power percentage changes from the baseline period [-0.3 -0.1] seconds. are shown with colors. The solid black line represents the average results of all ERP analyses from all participants (n = 48). (b) TMR elicited detectable reactivation. A linear classification shows a significant correct classification rate (CCR) compared to chance level of 0.25, and this effect is explained by a cluster (green shaded area, n = 48, p = 0.026) after correcting using cluster-based permutation. (c) Behavioral improvement is significantly higher for the cued sequence compared to the un-cued one (Wilcoxon signed-rank test, n = 41, p = 0.016, z = 2.42) indicating that TMR benefited the cued sequence. (d) Classification performance (CCR) correlated positively with memory improvement immediately after sleep (Spearman r = 0.45, p = 0.001, n = 48), the maximum classification CCR value for each participant was used, and a partial correlation controlling for pre-sleep behavioral performance also showed a significant correlation (Spearman r = 0.38, p = 0.009, n = 48).

TMR has been shown to elicit a distinguishable oscillatory pattern that is apparent in the time-frequency representation as well as ERP analysis. We looked at the TMR-elicited response in both time-frequency and ERP analyses using a similar approach to (Cairney et al., 2018). As presented in Figure 2a, EEG response showed an increase in theta band followed by an increase in sigma band, with the latter starting about one second after TMR onset. Furthermore, ERP analysis showed a small increase in ERP amplitude immediately after TMR onset, followed by a decrease in amplitude 500 ms after the cue. These findings demonstrate that TMR was effectively eliciting a response, thus confirming that our TMR cues were being processed by the brain.

### 3.2 Memory encoding activity during wake re-emerges in sleep after TMR cues

Several different methods for detecting memory reactivation have been adopted in the literature, some of which discriminated categories within sleep without the inclusion of wake (Cairney et al., 2018; Schönauer et al., 2017), while others selected features that showed high discrimination in wake (Wang et al., 2019). Our previous method directly relates wake and sleep activity using machine-learning classifiers, but those classifiers were trained on wake and tested on sleep (Abdellahi et al., 2023b). We have now improved our method so that the classifiers pay attention to features present in sleep that are related to reactivation. We did this by building a machine-learning model that was trained with the sleep data occurring after each TMR cue and tested during wakeful imagination of the trained task. This pipeline allows classifiers to weigh the features according to those present in sleep rather than weighing features according to those present in wake which could be dominated by effects that are absent from sleep. This also allows our linear classifiers to see the noise of sleep data represented in the within-class covariance.

Sleep data were used to train a linear discriminant analysis (LDA) classifier, and this classifier was applied to EEG data from wakefulness at every time point after the sound cues, giving a classification performance (correct classification rate, CCR) at every time point in wake. We trained LDA classifiers on our multi-class SRTT with each finger representing a class (4 classes in total, 2 fingers per hand). Classification performance was significantly above chance level (Fig. 2b, significant effect is explained by the cluster with the green shaded area, p = 0.026), and this shows that memory reactivation can be identified by our classification models.

Given that the task involved a sequence of trials in a fixed order, we were concerned that the brain might prepare responses in advance of the TMR cue. We, therefore, jittered the intertrial intervals between the TMR cues to eliminate this possibility. Trials, therefore, varied in durations by a maximum variation of one second between the shortest trial (2500 ms) and the longest trial (3500 ms). Given the uncertainty of the timing of reactivation, and the fact that it could sometimes happen after 2500 ms, we included all of the temporal information of the sleep data into our classification model by using time points as observations (see section 2).

### 3.3 TMR benefits cued sequence

Studies on the SRTT have shown a positive effect of TMR on consolidation (Cousins et al., 2014, 2016; Koopman et al., 2020). Here, we tested TMR-dependent consolidation by comparing SRTT performance between cued and un-cued sequences across the aggregated follow up sessions (see the section 2 for details). We found a benefit for the cued sequence as compared to the un-cued sequence across follow-up sessions (Wilcoxon signed rank test, n = 41, p = 0.016, z = 2.42, Fig. 2c). This shows the positive effect that TMR has on memory improvement. We also checked in individual sessions and found that the benefit is more prominent in the later follow-up sessions compared to the immediate follow-up, 24 hours follow-up: (Wilcoxon signed-rank test, n = 41, p = 0.141, z = 1.47), 10 days follow-up: (Wilcoxon signed-rank test, n = 41, p = 0.025, z = 2.235), and (Wilcoxon signed-rank test, n = 41, p = 0.0387, z = 2.067).

### 3.4 The strength of reactivation predicts memory consolidation

We wanted to test whether the elicited reactivation in sleep predicts the extent of TMR-dependent benefit right after sleep (24 hours). To this end, we conducted a Spearman correlation between the classification performance (CCR) and cueing benefit to reaction time right after sleep (reaction time for non-reactivated sequence – reaction time for reactivated sequence). This showed as strong positive relationship (Spearman r = 0.45, p = 0.001, n = 48), Figure 2d, supporting the idea that the reactivations detected by our classifiers underpin cueing benefit to reaction time. To examine the effects of pre-sleep performance during encoding, we also conducted a partial correlation between classification performance and improvement right after sleep (Spearman r = 0.38, p = 0.009, n = 48), see section 2. This revealed that the strength of reactivation positively predicts consolidation, supporting a functional role for our detected reactivation.

### 3.5 Memory reactivation in SWS is temporally compressed compared to wake

**Fig. 3.**
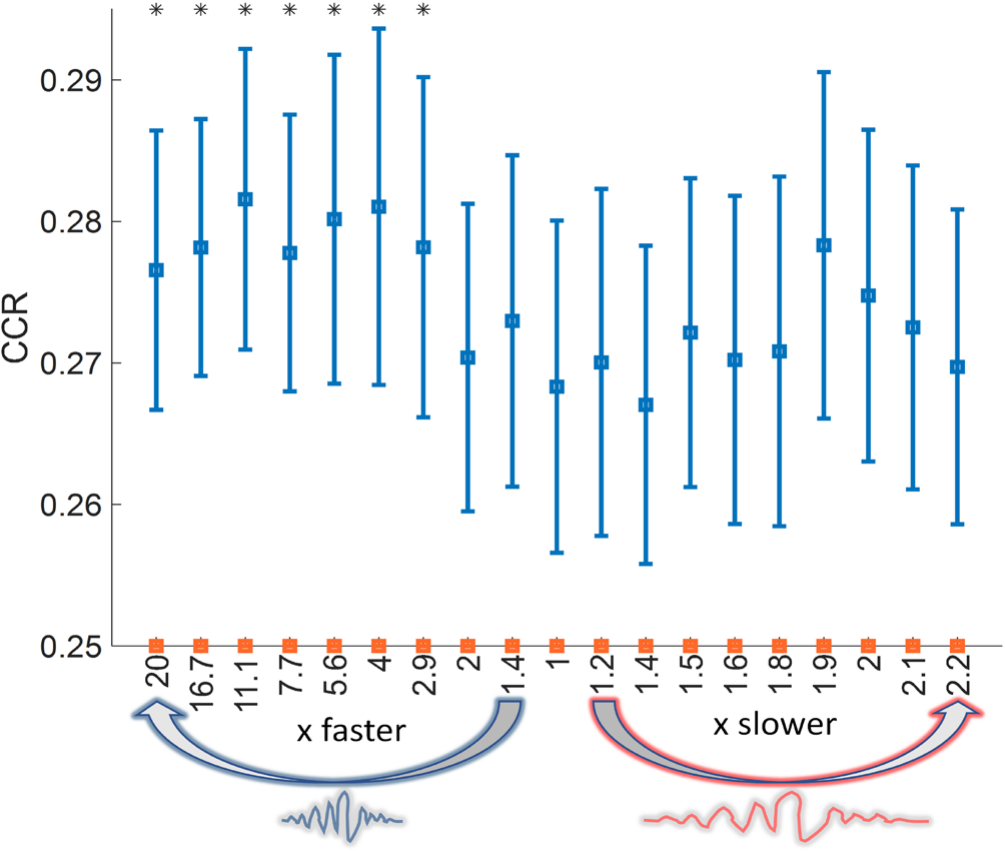
Analysis of temporal compression shows that reactivation is faster than wake pattern. The x-axis represents how much (x) faster or slower sleep reactivation was compared to wake, and the y-axis represents correct classification rate (CCR). Significant results (p < 0.05) are marked by asterisks.

We next tested whether sleep reactivation mimics the shape and duration of wake activation by performing an analysis of compression and dilation. In this analysis, we fixed the length of wake trials and progressively changed the length of sleep trials. We used a ratio (length of sleep trial / length of wake trial) to indicate the temporal ratio between sleep and wake duration. Thus, a ratio of less than one indicates compression, a ratio of exactly one indicates no compression or dilation, and a ratio of greater than one indicates dilation. For every ratio, we applied a sliding window approach where we took sleep windows according to the ratio and then resized them to match the length of wake trials. Afterward, we trained a classifier on sleep and tested it on wake to see if the sleep reactivation pattern was similar to wake at the given ratio (see section 2). Our results indicate that sleep reactivation is compressed compared to wake (Fig. 3), and this compression is 3 to 20 times faster than in wake.

### 3.6 Spindles hallmark reactivation

**Fig. 4.**
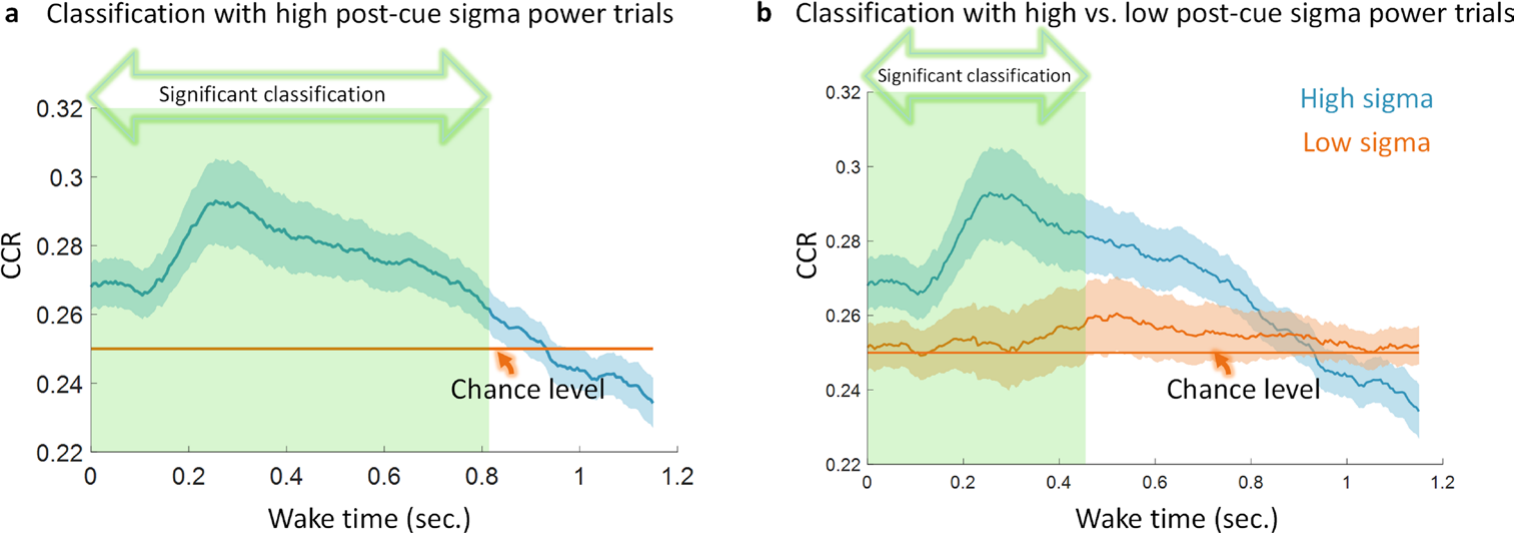
(a) Classification using sleep trials with high post-cue sigma power [11 16]Hz shows significant classification performance explained by a cluster (green shaded area, p = 0.001). CCR, correct classification rate. (b) Classification performance for trials with high post-cue sigma power compared to trials with low post-cue sigma power. This shows a significant difference explained by the cluster shaded in green (n = 48, p = 0.022).

We performed a median split on sigma power for the trials within each participant and we found that only trials with high post-cue sigma power showed evidence of reactivation (significant effect explained by a cluster p = 0.001, Fig. 4a) compared to chance level. This is in line with findings from Wang and colleagues, who examined TMR cued NREM reactivation during a similar task showed that trials with high post-cue sigma power [11 16] Hz were more likely to involve detectable reactivation (Wang et al., 2019). Both findings support the idea that high post-cue sigma power acts as a marker for reactivation. Interestingly, in our data, classification of these high-sigma trials was also significant when compared to classification using low sigma power trials (significant effect explained by a cluster p = 0.022, Fig. 4b).

## 4 Discussion

We examined the temporal characteristics of the reactivation of individual finger representations associated with an SRTT and provide evidence that reactivation happens faster than the original experience during wake. Our results also support earlier work suggesting that sleep spindles provide a marker of reactivation.

Some studies used only sleep data in their classification pipelines to show evidence for the reprocessing of memories during sleep (Cairney et al., 2018; Schönauer et al., 2017). Others performed within sleep classification with features selected from wake data (Wang et al., 2019) or by relating wake to optimal sleep lags (Belal et al., 2018). Here, we directly related neural responses in sleep to those during the imagery task in wake by training classification models on sleep observations and applying them on wake. This direct sleep-wake relationship means that our models will not mistakenly classify sleep EEG noise as reactivations. Thus, our linear classifiers can adapt to sleep and adjust their feature weights according to sleep patterns. This also enables our LDA models to see sleep noise represented by within-class covariance matrices and adapt to it. We successfully used this approach in classifying memory reactivation after TMR in human REM sleep (Abdellahi et al., 2023a); here, we use it for the first time in SWS along with PCA. To further elucidate the wake-sleep relationship, we used jittered inter-trial delays, thus preventing periodic oscillations from affecting the training of our models. Given that the finger-tapping task is a sequence, if we were to use fixed inter-trial delays the brain could have predicted and reactivated the contents of the upcoming TMR before it has actually been presented. Our jittered cues avoided this possible predictability. Trials from both cued and un-cued sequences were used when testing on wake which ensured that the classification was not derived from mere sound related patterns arising after cued items. We did not include a separate control night in this study; however, the correlation between classification strength and TMR -related behavioral improvement (Fig. 2d, Spearman r = 0.38, p = 0.009, n = 48) provides evidence that the classifier is detecting memory reactivation, as we would not expect such a correlation between ERP responses to the sounds delivered and TMR-related behavioral improvement. Also, our prior studies demonstrated that the time-domain features we used here are sufficient to successfully classify memory reactivation in this task (Abdellahi et al., 2023a, 2023b).

Several rodent studies have tackled the question of temporal compression of reactivation. Findings show that cell firing happens at a faster rate during replay compared to the original experience (Davidson et al., 2009; Euston et al., 2007; Ji & Wilson, 2007; Lee & Wilson, 2002; Nádasdy et al., 1999). Collectively, replay has been observed at different rates, ranging from 6 to 20 times faster than the waking experience. While previous studies of temporal compression have relied on neuronal recordings in non-human animals, here, we detect compression in large-scale neural coordination patterns measurable with EEG. This method allowed us to examine whether the temporal dynamics of memory reactivation, as reflected in cross-channel coordination and timing relationships, exhibited similar compression properties at the population level captured by scalp recordings. Our results are in-line with the literature, suggesting that reactivation happens at a rate that is around 3 to 20 times faster than wake. Importantly, reactivation is unlikely compressed 3-fold and 20-fold in the same trial. Compression factors could vary from one participant to another; however, we can say that our data generally support the idea of compressed reactivation on the EEG level.

It has been proposed that memories are transferred into a long-term store via repetitive reactivation (Diekelmann & Born, 2010). According to this view, there is a dialogue between the hippocampus and the neocortex wherein cortical SOs drive thalamo-cortical spindles. Ripples and their associated reactivations are nested in the troughs of these spindles, which emphasizes the importance of sleep spindles and ripples in the reprocessing of memories. Several papers have shown a direct relationship between memory reactivation and spindles in which spindles marked reactivation (Cairney et al., 2018; Wang et al., 2019). Moreover, Zhang and colleagues provided direct evidence that human memory replay happens during ripple events using intracranial EEG and similarity analysis (Zhang et al., 2018). We provide direct evidence of reactivation being marked by spindles, thus supporting the hypothesis that reactivation occurs during ripple events. This could explain why it is compressed in time. Indeed, the compression of 3 to 20 times observed in our data means that reactivations happen for a duration of 57 ms to 383 ms which could support the speculation that ripples can carry reactivations, since they are characterized by 50 to 100 ms of high-frequency activity (Ylinen et al., 1995). Despite the technical limitations of directly estimating ripple events in human cortical EEG, our temporal compression analysis helps to unravel the footprint of ripples and the impact they have on the temporal characteristics of the detected reactivation. Along with spindle analysis, this evidence fits well with the idea of spindle-ripple events as a hallmark for reactivation.

## 5 Conclusion

Our findings show that slow wave sleep reactivations of multiple memories are detectable in humans and occur faster than activation during the task. Furthermore, reactivation detectability positively correlated with memory improvement which reflects their functional significance. We also support prior work showing that spindles are hallmarks for reactivation. Overall, we describe new characteristics of reactivations and how they relate to wake. We also introduce a new method for detecting SWS reactivation by training classification models with sleep EEG and testing them on wake data.

## Supplementary Material

Supplementary Material

## Data Availability

Data and scripts are available on OSF and GitHub along with detailed instructions on running different analyses and system requirements: https://osf.io/byvcg/?view_only=9b149e0387814bf1a6fca692f90e9167 and https://github.com/MahmoudAbdellahi/Targeted-memory-reactivation-elicits-temporally-compressed-reactivation-linked-to-spindles. Participants’ private identifications are all anonymized.
