# Supplementary material for: Targeted memory reactivation elicits temporally compressed reactivation linked to spindles
Source: Imaging Neurosci (Camb). 2026 Feb 3;4:IMAG.a.1123. doi: 10.1162/IMAG.a.1123 (PMC12869320; doi:10.1162/IMAG.a.1123)
Supplement: Supplementary Material [file IMAG.a.1123_supp.pdf]

Supplementary material

Targeted memory reactivation elicits temporally compressed reactivation linked to spindles

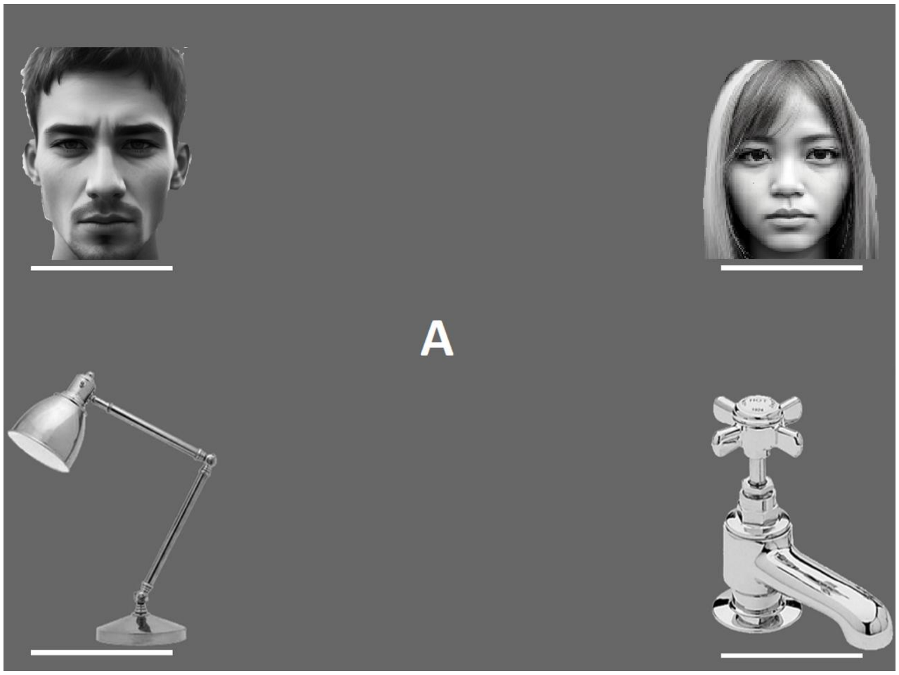

Supplementary figure 1: Illustration of the four images that appeared in the task: two faces and two objects. Faces in this illustration were artificially generated using AI.

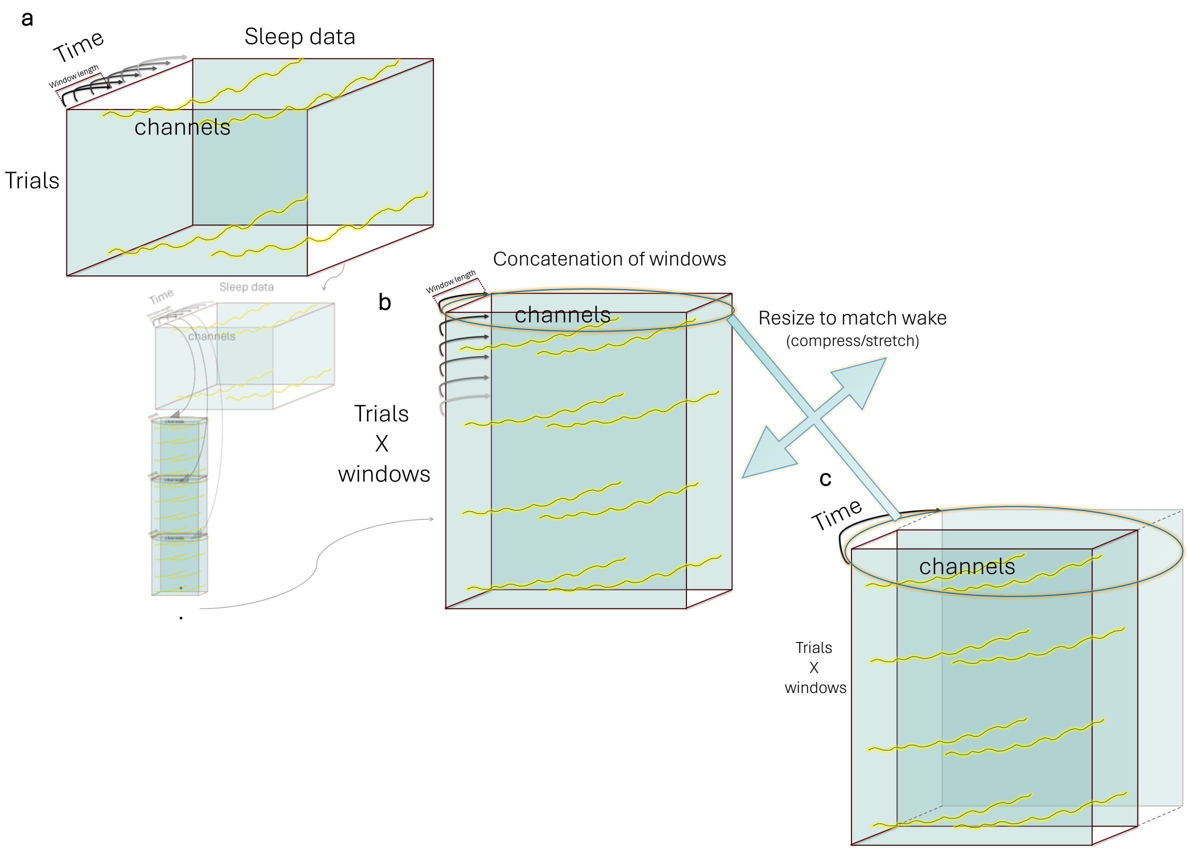

Supplementary figure 2: a) Illustration of the temporal compression analysis, each trial from sleep data was divided into several windows across time depending on the tested compression ratio. **b)** Different windows are then concatenated in the trial dimension resulting in window length becoming the time dimension of sleep trials and windows x original trials becoming the new trials dimension. **c)** Consequently, all of those new trials are temporally resized from ‘window length’ to the duration of wake trials.
